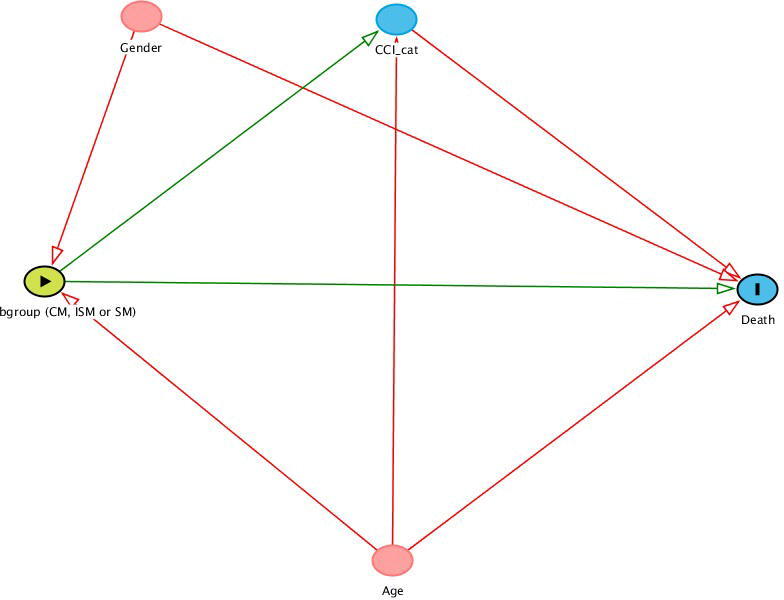# How “benign” is cutaneous mastocytosis? A Danish registry-based matched cohort study

**DOI:** 10.1016/j.ijwd.2020.05.013

**Published:** 2020-06-01

**Authors:** Line Kibsgaard, Mette Deleuran, Carsten Flohr, Sinéad Langan, Anne Braae Olesen, Christian Vestergaard

**Affiliations:** a Department of Dermatology, Aarhus University Hospital, Aarhus, Denmark; b Unit for Population-based Dermatology Research, St John’s Institute of Dermatology, Guy’s & St Thomas’ NHS Foundation Trust and Kings College London, London, United Kingdom; c Faculty of Epidemiology & Population Health, London School of Hygiene and Tropical Medicine, London, United Kingdom

**Keywords:** Epidemiology, Mastocytosis, Comorbidity-adjusted analyses, Survival analyses, Danish National Patient Registry

## Abstract

**Background:**

There are limited estimates of the incidence rates (IRs) of mastocytosis, and only a few studies have addressed the long-term consequences of living with these diagnoses. Previous reports have shown that systemic mastocytosis is associated with leukemic transformations and an increased risk of death as opposed to cutaneous mastocytosis (CM) and indolent systemic mastocytosis (ISM), which have benign diagnoses with life expectancy rates similar to those of the background population.

**Objective:**

This study aimed to analyze the incidence and mortality of mastocytosis.

**Methods:**

A population-based matched cohort study of patients with mastocytosis between 1 January 1, 1977 and 31 December 31, 2014 was identified from the Danish National Health Registries. IRs of CM, ISM, and pediatric mastocytosis were highlighted. Survival estimates were compared with those of a healthy background population, using a Cox proportional hazard model.

**Results:**

A total of 1461 patients with mastocytosis were identified. The annual IR of overall mastocytosis was 1.1 per 100,000 person years (95% confidence interval [CI], 1.0–1.2). Among children, the IR was 1.8 per 100,000 person years (95% CI, 1.6–2.1). The prevalence of any comorbidity was twice as high among patients with mastocytosis compared with the population without mastocytosis (odds ratio: 2.1; 95% CI, 1.8–2.5). The Charlson Comorbidity Index–adjusted mortality among adult patients with mastocytosis was HR_Cutaneous Mastocytosis_ 1.2 (95% CI, 0.8–1.9), HR_Indolent Systemic Mastocytosis_ 1.9 (95% CI 1.4–2.5), and HR_Systemic Mastocytosis_ 4.2 (95%, CI 1.9–9.4), respectively.

**Conclusion:**

Based on an entire nation, with free health care at the point of access, we estimated an annual IR of mastocytosis and its subgroups. We discovered that patients with ISM had an increased risk of death compared with the general population. Our data supported the overall benign nature of CM diagnosed after age 2 years.

## Introduction

Mastocytosis is a heterogeneous group of rare mast cell disorders that may affect both children and adults. Mastocytosis is divided into three major disease categories: cutaneous mastocytosis (CM), systemic mastocytosis (SM), and localized mast cell tumors (Valent et al., 2017). The first case of CM was reported in 1869 and the first case of SM in 1949, but there was no international consensus on how to differentiate CM from SM or vice versa until 2001 (Valent et al., 2001).

Pediatric cases of mastocytosis are almost always benign and in the category of CM, where spontaneous remission is a well-known phenomenon. However, a few cases of pediatric SM have been reported with onset before the age of 2 years and mostly as cases of diffuse CM, which is associated with anaphylaxis, gastrointestinal bleeding, and bullous erythrodermia (Ben-Amitai et al., 2005, Burnett et al., 2007, Ghiasi et al., 2011, Lange et al., 2013, Meni et al., 2015). Aggressive subtypes of mastocytosis are thus reported in infants and in patients aged ≥60 years (Bodemer et al., 2010, Escribano et al., 2009). More than 30% of those with adult-onset mastocytosis will develop systemic involvement over time (Valent et al., 2007). Age at onset and clinical subtype are thus prognostic factors in the clinical assessment of patients with mastocytosis.

SM is associated with leukemic transformation and an increased risk of death (Pardanani, 2015), but patients with indolent SM (ISM) reportedly have life expectancy rates that are similar to those of the background population (Escribano et al., 2009). In 2016, a subcategory of ISM was finally accepted: smoldering SM (SSM). These patients were observed to have a less favorable prognosis than those with ISM, but a superior prognosis when compared with patients with aggressive SM and mast cell leukemia (Table 1; Arber et al., 2016). Epidemiologic studies on long-term outcomes in larger mastocytosis cohorts are limited and biased toward aggressive subtypes of SM (tertiary center cases).

**Table 1:** The World Health Organization classification of mastocytosis in 2016 (Valent et al., 2017).

**Cutaneous mastocytosis (CM)**
Maculopapular CM (urticaria pigmentosa)
Diffuse CM
Mastocytoma of the skin
**Systemic mastocytosis (SM)**
Indolent SM
Systemic SM
SM with clonal hematologic non-mast cell-lineage disease
Aggressive SM
Mast cell leukemia
Mast cell sarcoma

The scope of this study was to examine the incidence of mastocystosis in Denmark between January 1977 and December 2014. We investigated the long-term survival of people with CM, SM, and ISM adjusted for sex and age as well as competing comorbidities using the Charlson Comorbidity Index (CCI) score.

## Methods

### Design

In this population-based cohort study, patients with mastocytosis between January 1, 1977 and December 31, 2014 were identified in the Danish National Health Registries. The study was approved by the Danish Health Authority (project no. 3-3013-338/1) and reported in accordance with the Reporting of Studies Conducted using Observational Routinely Collected Data Statement (Benchimo et al., 2016, Vandenbroucke et al., 2007).

### Data sources

#### Civil registration system

All live-born citizens in Denmark are assigned a personal social security number, the Civil Personal Register number. This number is included in all Danish health and public registries and was used to facilitate the extraction of individual diagnoses and vital status of the participants in the present study (Schmidt et al., 2014). Information is updated on a daily basis.

#### Danish National Patient Registry

The Danish National Patient Registry contains information on all hospital admissions from January 1, 1977. For each admission, a record of diagnoses is registered, with the primary (or A-) diagnosis being the cause of admission. Existing or adjacent diseases are registered as secondary (or B-) diagnoses (Schmidt et al., 2015). From January 1, 1995, diagnoses from all outpatient and emergency room visits were added. The International Classification of Diseases (ICD), version 8, codes were used until 1994 and version 10 (ICD10) codes thereafter. Diagnostic coding is a mandatory procedure in all discharge summaries and is conducted by physicians exclusively.

#### Danish Pathology Registry

The Danish Pathology Registry was established in 1997 as a coorporation between the Danish Society of Pathologists and the National Board of Health Data. This registry contains diagnoses (SNOMED diagnoses) as assessed by trained pathologists on all cell and tissue samples in primary, secondary, and tertiary health care settings. The registry contains data on almost 100% of pathologic samples (Bjerregaard and Larsen, 2011).

### Study population

Diagnostic codes for mastocytosis were used to screen and identify people with mastoctytosis from either of two registries: Danish National Patient Registry or Danish Pathology Registry (Supplementary A). Baseline for each individual refers to the first date in an individual’s record on which the diagnosis of mastocytosis was noted. Ten age- and sex-matched individuals, per patient with mastocytosis, entered the study on the same date. A cohort of 1461 patients with mastocytosis and 14,610 people without mastocytosis were compared in terms of demographic characteristics and comorbidities at baseline. Patients with mastocytosis were identified from January 1, 1994 to December 31, 2014 and categorized as CM, ISM, or SM. Age was categorized as infant (0–1 years of age), child (2–15 years of age), young adult (16–60 years of age), and older adult (age ≥ 60 years) for descriptive and analytical purposes. The ICD version 8 codes did not allow for us to distinguish between subgroups of mastocytosis; thus, the epidemiologic analyses were restricted to the ICD10 period.

### Comorbidity

The CCI was introduced in 1987 as a new method to assess the risk of death from comorbid disease categories in longitudinal studies. The index consists of 19 chronic diseases that are weighted in proportion to severity (Charlson et al., 1987). The CCI is widely used to assess comorbidity among adults with a wide spectrum of diseases (Quan et al., 2011). In this study, baseline comorbidity was based on prior and present diagnoses included in the CCI (Supplementary B). Diagnoses were recorded as a binary (0 or CCI weight) as well as categorized variable (low [0], moderate [1–2], or high [≥3]). The CCI has not been validated for use in pediatric populations, but children were not included in the comorbidity and CCI-adjusted mortality analyses.

### Statistical analysis

Incidence rates (IRs) of childhood (<16 years of age) mastocytosis were assessed during the complete study period, and we calculated the Danish childhood prevalence as of December 31, 2014 (Statistics Denmark, 2016).

Statistical analyses were defined beforehand and performed using STATA, version 13.1 (Stata Statistical Software). Prospective analyses were conducted, following the cohort from staggered entry until the time of death, loss to follow-up, or end of follow-up (December 31, 2014), whichever came first. Influent covariates were assessed on the basis of the literature and a preanalytic directed acyclic graph (Supplementary C). Age was strongly associated with the CCI, and adjustment of either sex and age or sex and CCI appeared to deliver the most reliant results. We used a logistic regression model to compare baseline CCI scores (0 or >0).

We identified all deaths in patients with mastocytosis and matched individuals. The Kaplan-Meier model was used to compare survival probabilities at 1 and 5 years after entering the study. A Cox regression model adjusting for age (categorical) and sex was used to compare the risk of death. Stratified analyses were undertaken to determine whether there was effect modification by age. Assumptions on proportional hazards were fulfilled as evaluated from graphic assessments of log–log plots.

## Results

A total of 1300 patients with mastocytosis were identified during the ICD10 period. Of these, 25 patients were excluded, either because the inclusion date came after the registered date of death (n = 1) or because the ICD10 diagnoses were registered before the official date of the ICD10 implementation (n = 24). Thus, IRs and mortality analyses were based on 1275 cases of mastocytosis. The study population was followed for a median of 9 years (interquartile range [IQR], 4–17 years) and pediatric cases for a median of 8 years (IQR, 4–14 years).

Of the 1436 patients with mastocytosis (Table 2), 1005 (70%) were adults with a median age at onset of 48 years (IQR, 36–61 years). The male-to-female ratio was 2:3 in adult cases. More men than women were classified as SM cases (73%), excluding cases of ISM. A total of 431 patients (30%) were diagnosed during childhood, and 273 of these patients (63%) were diagnosed within the first 2 years of life. The median age at onset was 10 months among infants (IQR, 6–13 months) and 4.5 years in the child population (IQR, 2.8–8.0 years). The male-to-female ratio among children was 3:2, and the majority of pediatric cases had diagnoses of CM (95%).

**Table 2:** Characteristics of patients registered with diagnosis of mastocytosis in Denmark from January 1, 1977 to December 31, 2014.

Characteristics	All mastocytosis ICD8 + ICD10 (1977–2014) N = 1436	Cutaneous Mastocytosis ICD10 (1994–2014) n = 856	Indolent systemic mastocytosis ICD10 (1994–2014) n = 393	Systemic Mastocytosis ICD10 (1994–2014) n = 26	Urticaria pigmentosa ICD8 (1977–1993) n = 161
Age categories, n (%), y					
0–1	273 (19)	231 (27)	12 (3)	0 (0)	30 (19)
2–14	158 (11)	128 (15)	4 (1)	1 (4)	25 (16)
≥15	1005 (70)	497 (58)	377 (96)	25 (96)	106 (66)
Mean age (±SD), y	35 (±26)	28 (±24)	52 (±21)	61 (±16)	29 (±24)
Median age, y (25/75 percentiles)					
0–1	0.8 (0.5/1.1)	0.7 (0.5/1.1)	0.7 (0.5/0.8)	NA	1.0 (0.6/1.4)
2–14	4.5 (2.8/8.0)	4.6 (2.8/8.4)	5.3 (3.1/7.6)	6.5 (NA)	4.4 (3.2/6.5)
≥15	47.5 (36.4/61.4)	45.1 (33.7/56.4)	51.3 (40.2/69.5)	65.0 (55.4/72.5)	45.4 (35.4/54.8)
Year of diagnosis					
1977–1993	161 (11)	9 (1)	12 (3)	0 (0)	161 (100)
1994–2000	398 (28)	205 (24)	181 (46)	9 (35)	NA
2001–2014	877 (61)	642 (75)	200 (51)	17 (65)	NA
Follow-up, y (25/75)*					
Median age at	49 (22/68)	37 (14/58)	64 (50/79)	71 (58/84)	59 (34/77)
Median years of	8.6 (3.3/16.0)	7.5 (3.7/13.2)	6.9 (1.1/15.5)	0.8 (0.2/5.3)	27.6 (22/33)
Sex					
Female	761 (53)	462 (54)	208 (53)	7 (27)	84 (52)
Male	675 (47)	394 (46)	185 (47)	19 (73)	77 (48)
CCI scores at baseline					
Low (0)	1217 (83)	776 (90)	273 (67)	8 (31)	160 (99)
Moderate (1–2)	198 (14)	79 (9)	103 (25)	15 (58)	198 (14)
High (≥3)	46 (3)	12 (1)	31 (8)	3 (11)	46 (3)
Mean CCI (95% CI)†	0.36 (0.31–0.41)	0.17 (0.13–0.20)	0.81 (0.67–0.96)	1.69 (1.03–2.36)	0.01 (-0.01–0.02)
Bone marrow biopsy					
Performed in cases	275 (19)	148 (17)	105 (26)	13 (50)	9 (6)
Children	6 (1.4)	5 (1.4)	0 (0)	0 (0)	1 (1.5)
Adults	269 (26)	143 (28)	105 (27)	13 (52)	8 (8)
Diagnostic units					
Dermatology	574 (40)	413 (48)	102 (26)	2 (8)	57 (41)
Allergology	101 (7)	78 (9)	21 (5)	1 (4)	1 (1)
Paediatrics	114 (8)	77 (9)	0 (0)	0 (0)	37 (26)
Hematology	99 (7)	41 (5)	42 (11)	4 (15)	12 (4)
Emergency units	14 (1)	8 (1)	6 (1)	0 (0)	0 (0)
Internal medicine	144 (10)	48 (6)	42 (11)	9 (35)	15 (23)
Surgery	185 (13)	59 (7)	114 (29)	3 (12)	9 (4)
Pathology‡	193 (13)	125 (15)	61 (15)	7 (27)	0 (0)
Other	12 (1)	7 (1)	5 (1)	0 (0)	0 (0)

CI, confidence interval; CCI, Charlson Comorbidity Index; ICD, International Classification of Diseases; SD, standard deviation.

Patients identified by the ICD10 diagnostic code system (1994–2014) were divided into cutaneous, indolent systemic, and systemic mastocytosis cohorts. Patients identified from 1977–1993 relied on the ICD8 diagnostic code system. Figures are numbers with appertaining percentages. Continuous variables are presented with appertaining SDs or 25/75 percentiles.

* For the purpose of survival analysis, patients were followed from time of diagnosis until death, end of follow-up (December 31, 2014), or loss to follow-up.

† The prevalence of chronic comorbidities was compared to a sex- and age-matched nonmastocytosis group with mean CCI = 0.14 (0.13–0.15).

‡ Mastocytosis diagnosis based exclusively upon diagnosis from the Danish National Pathology Register.

### Incidence

The annual IR of mastocytosis was 1.1 cases per 100,000 person years (95% confidence interval [CI], 1.0–1.2). There was no trend in the frequency of diagnoses over time (data not shown). The annual IR of CM was 0.75 cases per 100,000 person years (95% CI, 0.7–0.9), whereas the IR of ISM was 0.4 cases per 100,000 person years (95% CI, 0.3–0.5). The annual IR among children (age < 16 years) was 1.8 cases per 100,000 person years (95% CI, 1.6–2.1), and the prevalence of pediatric mastocytosis as of December 31, 2014 was 2.5 cases per 10,000 children.

### Comorbidities

Adult ISM and SM cases had a higher baseline prevalence of chronic comorbidities (Table 3). In the multivariate analysis model, CCI-adjusted hazard ratios (HRs) did not differ significantly from the HRs from the age-adjusted analyses. Comorbidities with a higher prevalence were metastatic cancers with unknown localization (OR_MAST_ 9.3; 95% CI, 5.1–17.1), chronic pulmonary diseases (OR_MAST_ 1.7; 95% CI, 1.1–2.5), and connective tissue diseases (OR_MAST_ 2.3; 95% CI, 1.3–4.0).

**Table 3:** ORs and 95% CIs on comorbidities at baseline in adult (≥15 years of age) patients with mastocytosis compared with age- and sex-matched unexposed population (n = 8920).

Mastocytosis	No.	Crude risk of CCI ≥ 1 OR (95% CI)	Adjusted risk of CCI ≥ 1 OR (95% CI)*	Adjusted risk of CCI ≥ 1 OR (95% CI)†
Mastocytosis overall	891	2.10 (1.80–2.45)	2.44 (2.06–2.90)	1.63 (1.35–1.97)
Cutaneous mastocytosis	489	1.26 (0.98–1.62)	1.50 (1.15–1.94)	1.57 (1.19–2.08)
Indolent systemic mastocytosis	377	3.51 (2.82–4.39)	3.47 (2.75–4.39)	1.83 (1.38–2.41)
Systemic mastocytosis	25	17.37 (7.24–41.69)	12.31 (4.89–30.99)	1.63 (0.63–4.19)

CI, confidence interval; OR, odds ratio.

Charlson Comorbidity Index scores (CCI) were dichotomised into 0 or ≥1.

* Adjusted for age (> or <60 years of age) and gender.

† Sensitivity analysis omitting diagnoses of lymphoma, leukaemia and non-metastatic solid tumour categories (C00-C75, C91-C95, C81-C85, C88, C90, C96).

### Survival

During 125,115 person years of follow-up, a total of 1405 persons (10%) died, 186 (15%) from the patient group and 1219 (10%) from the comparison group. Four of seven childhood deaths occurred in patients with mastocytosis (findings not presented to preserve confidentiality). A total of 248 persons (1.8%) were censored due to emigration or loss to follow-up. The relative 5-year survival was 1.0 (95% CI, 1.0–1.0) in the adult CM group, 0.8 (95% CI, 0.8–0.9) in the ISM group, and 0.4 (95% CI, 0.3–0.7) in the SM group. The raw survival data on CM cases diagnosed between 1977 and 2014 were similar to those of the background population and showed a tendency toward a protective effect (data not shown).

**Fig. 1:**
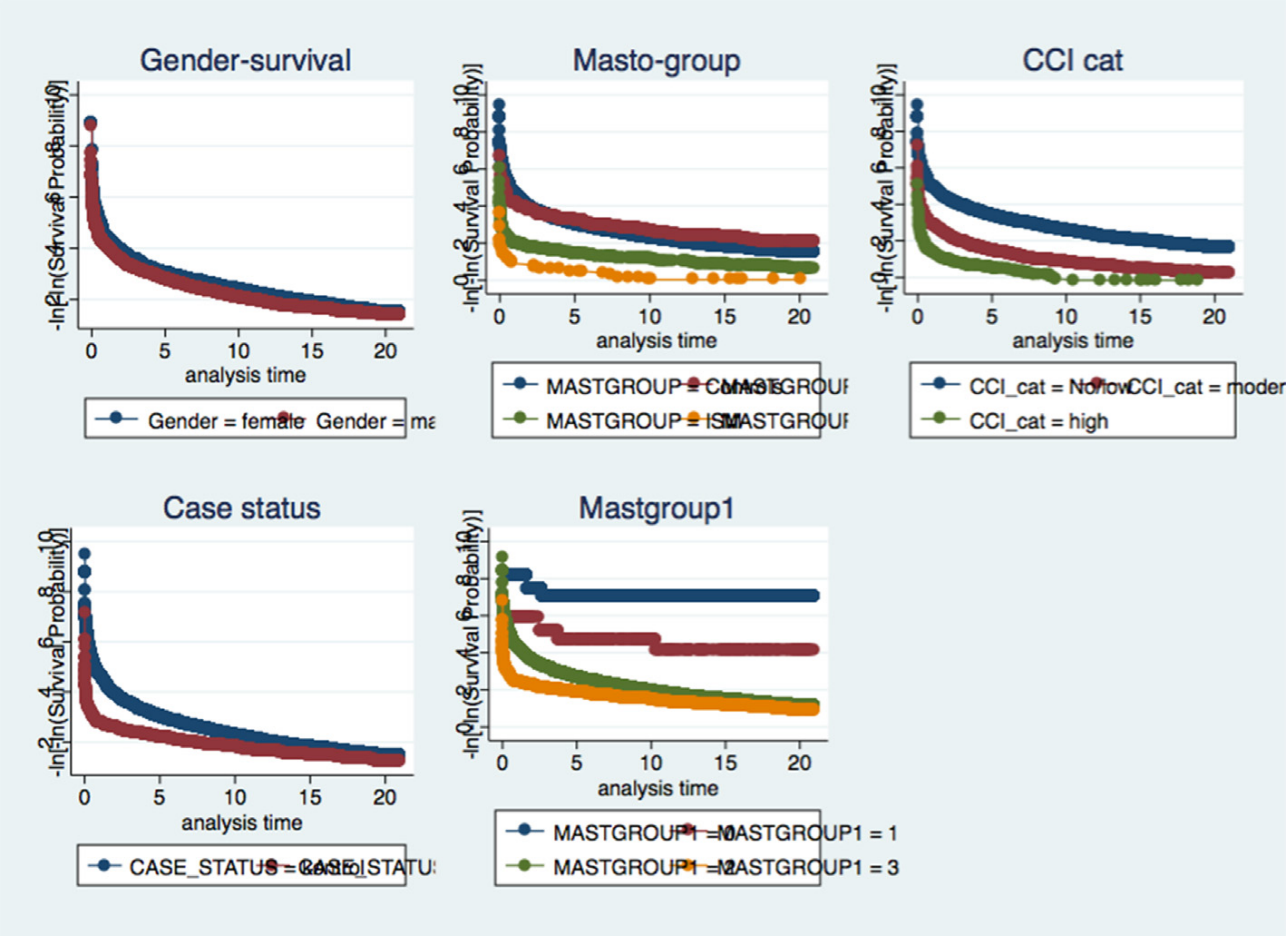
Confounding variables and their influence during a period of twenty years of follow up.

Overall, patients with mastocytosis had a higher mortality rate compared with the background population (Table 4). When stratified by CM, ISM, and SM, the age- and sex-adjusted HRs were all significantly higher than within the comparison group. The decreased survival over time is illustrated in Fig. 1. Age was an effect modifier; thus, estimates were reported in relevant age strata. When adjusting for baseline CCI, the survival of patients with CM and ISM moved toward one (Table 4; columns 2–3).

**Table 4:** Crude and adjusted HR of death among patients registered with diagnosis of mastocytosis during the period from January 1, 1994 to December 31, 2014 (n = 1275).

Mastocytosis	Outcome (n)*	Crude risk of death HR (95% CI)	Adjusted risk of death HR (95% CI)†	Adjusted risk of death HR (95% CI)‡
**Mastocytosis all**	**186**	**1.64 (1.40**–**1.91)**	**2.13 (1.83**–**2.49)**	**1.71 (1.46**–**2.01)**
**CM**	**55**	**0.71 (0.54**–**0.93)**	**1.34 (1.02**–**1.76)**	**0.80 (0.61**–**1.06)**
0– 1 year		0.14 (0.04–0.42)	**15.23 (2.54**–**91.12)**	NA
2–15 years		0.07 (0.01–0.52)	10.49 (0.66–167.65)	NA
16–60 years		0.53 (0.34–0.83)	1.33 (0.84–2.12)	1.26 (0.79–2.01)
≥60 years		6.02 (4.24–8.57)	1.24 (0.87–1.77)	1.15 (0.81–1.64)
**ISM**	**114**	**3.24 (2.67**–**3.93)**	**2.59 (2.14**–**3.14)**	**1.53 (1.25**–**1.86)**
0–1 year		NA	NA	NA
2–15 years		NA	NA	NA
16–60 years		0.95 (0.65–1.39)	2.21 (1.48–3.28)	1.97 (1.33–2.92)
≥60 years		13.86 (11.15–17.24)	2.73 (2.19–3.41)	1.80 (1.42–2.27)
**SM**	**17**	**18.42 (11.41**–**29.75)**	**6.60 (4.08**–**10.68)**	**5.58 (3.44**–**9.07)**
0–1 year		NA	NA	NA
2–15 years		NA	NA	NA
16–60 years		13.18 (4.93–35.21)	37.07 (13.79–99.62)	26.01 (9.66–70.01)
≥60 years		31.80 (18.39–54.97)	5.48 (3.17–9.51)	3.08 (1.76–5.36)

CI, confidence interval; CM, cutaneous mastocytosis; HR, hazard ratio; ISM, indolent systemic mastocytosis; SM systemic mastocytosis.

Estimates are presented in clinically relevant age strata. HRs refer to the unexposed population (n = 12,760).

* The number of outcomes (deaths) within age strata were omitted in agreement with the national laws on protection of data referable to an individual level.

† Adjusted for age (categorical) and sex.

‡ Adjusted for Charlson Comorbidity Index score (categorical) and sex.

## Discussion

To our knowledge, this is the largest cohort of patients with mastocytosis ever presented. Follow-up was obtained over a period of almost 40 years, and the data were derived from one of the oldest and most complete population registries in the world.

### Epidemiology

Our findings corroborate those of a Danish study that estimated an annual IR of SM, including ISM, of 0.89 per 100,000 person years (Broesby-Olsen et al., 2016). A Dutch cross-sectional study found a prevalence of adult ISM of at least 13 cases per 100,000 person years (van Doormaal et al., 2013). The corresponding point prevalence in our case was 18 per 100,000 person years.

We discovered that 62% of all pediatric patients with mastocytosis were diagnosed before 2 years of age. This percentage was considerably lower than the 90% reported by Meni et al. (2015), but because our results are based on unselected register-based information, we believe that the percentage of infantile onset is closer to 60% than to 90% of all pediatric cases, which is in line with the findings of Schena et al. (2016).

### Survival

Patients with ISM and SM had a significantly increased risk of death, which was detectable after 1 year of follow-up. Considering the number of CM diagnoses in the adult population (n = 497), there is a chance of historical misclassification of ISM cases into an adult CM category, as pointed out in the study by Broesby et al. (2016), where adult cases of urticaria pigmentosa were consequently categorized as probable ISM. We observed an increased hazard of death in those with ISM and SM in all analyses.

Several studies emphasize the inferior survival of adult patients with SM, but only two studies address the decreased survival in the ISM patient category (Cohen et al., 2014, Pardanani, 2010). In the first study, the inferior survival among patients with ISM was downsized as a result of the misclassification of SM cases into the ISM category. The latter study acknowledged a lower survival in patients with SSM, a rare and slowly progressing subvariant of ISM that was mentioned for the first time by Valent et al. (2001). There is reason to believe that historical ISM cases may have progressed into SSM during the follow-up period, which may explain the observed decreased survival of patients with ISM in our study.

SSM was not included as an independent disease category until the 2016 World Health Organization Disease Classification; thus, it was impossible for us to redefine ISM cases into a new disease category not defined at the time of the data collection process. Therefore, survival estimates of patients with ISM in the present study should be interpreted with caution, although some adult patients with CM may have been misclassified as ISM cases or people who had SSM may have been included in the ISM category to reduce the survival of this group. Subgroup-specific HRs were robust to sensitivity analyses regrouping patients with CM who were reclassified as ISM or SM during follow-up.

In a literary review, 2.9% of pediatric patients with mastocytosis were reported to have a fatal outcome (Meni et al., 2015). In this study, children with SM were overrepresented, and no control group was included for comparison. In the present study, patients with childhood mastocytosis had a significantly different survival profile compared with children in the background population. However, the comparison was based on three versus four deaths among children of 0–1 year of age (HR: 15.23; range, 2.54–91.12), and there is a substantial risk that this estimate could have been related to chance, supported by the width of the CIs. Another explanation could be that these children may have had the rare diffuse CM; this diagnosis was not observed in this dataset.

### Strengths

This study was designed to overcome some of the methodological limitations of previous epidemiologic mastocytosis studies. The study was based on almost all Danish patients with mastocytosis over an extensive period of time. The cohort was identified from Danish National Health Registries, which offer unique possibilities for epidemiologic research on unselected groups with a high validity overall (Bjerregaard and Larsen, 2011, Schmidt et al., 2014, Schmidt et al., 2015, Zuberbier et al., 2009). To include the full spectrum of mastocytosis and minimize the risk of selection bias, two international diagnostic code systems (ICD and SNOMED) and registries were used for patient identification.

We used a matched cohort design to ensure adequate groups for comparison and adjusted analyses when assessing the prevalence of comorbidities or death as a prospective outcome within each subgroup of mastocytosis. The CCI has not been validated for use in mastocytosis but is recognized as a valid prognostic tool in less rare disease categories. Contrary to the study by Broesby et al. (2016), the effect of a weighted baseline CCI was tested as an influent variable in our prospective survival analyses. Our data suggest that adults with CM have a normal life expectancy rate, especially if data are adjusted for the effect of existing baseline chronic comorbidities.

### Limitations

Although the use of register-based data minimizes the risk of selection bias, the risk of a misclassification of the acquired data is present. The distribution of ISM/SM was consistent with the literature from 2014 (94% vs. 90%-95% as proposed by Brockow [2014]). We were limited by a relatively small number of patients in each subgroup, and age-stratified HRs especially should be interpreted with caution. The CCI has not been validated for use in children, nor has it been validated for descriptive purposes, although it has been used as such in a number of epidemiologic studies of Danish origin. The results from this study are only representative for mastocytosis cases of a certain severity, and the incidence (especially in the child population) is most likely underestimated. The study population was predominantly Caucasian, and our results might not be generalizable to countries with a population composition that differs significantly from that of Scandinavian countries. The effect of anaphylaxis and severe allergies were not integrated as separate variables in our survival analyses but might explain the higher mortality among some mastocytosis patients.

The increased mortality in our adult ISM category might be interpreted as a consequence of a historical misclassification and supports the integration of SSM as a future separate disease category. We therefore suggest that standardized outpatient visits could be limited to patients with CM whose disease history includes suspicion of a systemic involvement (relapsing and unexplained cases of anaphylaxis, anaphylaxis due to wasp sting, and/or pulsating abdominal pains) and patients who experience troublesome symptoms with the need for continued treatment and expert guidance.

## Conclusion

Based on historical Danish registry data, we discovered a frequency of 65 new Danish cases of mastocytosis per year. More children than adults were diagnosed with mastocytosis (childhood-to-adult ratio was at least 1.6).

Over time, patients with ISM had a mortality rate that was twice that of the background population. When adjusting for CCI, the survival of patients with CM and ISM moved toward one, which suggests that CM and ISM may be benign diagnoses in isolation and that the observed inferior survival may be a reflection of existing but unrecognized chronic diseases that are diagnosed when patients enter a hospital setting. A possible explanation for the persisting increased mortality observed in ISM, even after adjusting for the categorical CCI, could be that this group includes patients with SSM who are now known to have an increased mortality. The decreased survival of infant mastocytosis cases is difficult to interpret because the analysis was underpowered. Our study highlights the need for precise diagnostic coding of these diseases to enable studies of their epidemiology and natural history.

## Conflict of Interest

None.

## Funding

NA.

## Study Approval

The author(s) confirm that any aspect of the work covered in this manuscript that has involved human patients has been conducted with the ethical approval of all relevant bodies.

## Financial Disclosures

Carsten Flohr holds a UK National Institute for Health Research (NIHR) Career Development Fellowship (CDF-2014-07-037). Sinéad Langan was supported by a Welcome senior research fellowship in clinical science (205039/Z/16/Z), as well as by Health Data Research UK (grant no. LOND1), which is funded by the UK Medical Research Council, Engineering and Physical Sciences Research Council, Economic and Social Research Council, Department of Health and Social Care (England), Chief Scientist Office of the Scottish Government Health and Social Care Directorates, Health and Social Care Research and Development Division (Welsh Government), Public Health Agency (Northern Ireland), British Heart Foundation and Welcome Trust. Sinéad Langan previously held an NIHR Clinician Scientist Fellowship (NIHR/CS/010/014). The views expressed in this publication are those of the authors and not necessarily those of the NHS, Welcome, the NIHR, or the UK Department of Health.

## Figures and Tables

**Supplementary figure 1:**