# Supplementary material for: How “benign” is cutaneous mastocytosis? A Danish registry-based matched cohort study
Source: Int J Womens Dermatol. 2020 Jun 1;6(4):294–300. doi: 10.1016/j.ijwd.2020.05.013 (PMC7522902; doi:10.1016/j.ijwd.2020.05.013)
Supplement: Supplementary data 1 [file mmc1.docx]

|  | **ICD codes**  **(Danish National Patient Registry)** | **SNOMED codes**  **(Danish Pathology Registry)** | **Corresponding**  **WHO-classification diagnoses** |
| --- | --- | --- | --- |
| **Cutaneous mastocytosis (ICD10)** | DQ822A: Urticaria pigmentosa  DQ822B**:** Telangiectasiae macularis eruptiva perstans  DQ822: Mastocytosis (excludes malignant mastocytoma)  DD470A: Unspecified mast cell tumour  DD470 & age ≤ 15: Uncharacterised neoplasia of mast cell or histiocytic origin^1^ | M97401: Mastocytoma | Maculopapular cutaneous mastocytosis (MPCM)  Diffuse Cutaneous Mastocytosis (DCM)  Cutaneous Mastocytoma (Mast) |
| **Indolent systemic mastocytosis**  **(ICD10)** | DD470B: Indolent systemic mastocytosis  DD470 & age > 15: Uncharacterised neoplasia of mast cell or histiocytic origin^2^ | M974A1: Indolent systemic mastocytosis  M97411: Mastocytosis unknown if benign or malignant^3^ | Indolent systemic mastocytosis (ISM) |
| **Systemic mastocytosis**  **(ICD10)**  **Urticaria Pigmentosa**  **(ICD8)** | DD470C: Systemic mastocytosis with an associated clonal haematological non-mast cell lineage disease  DC943: Mast cell leukaemia  DC962: Malignant mast cell tumour  Dia75722: Urticaria Pigmentosa | M97413: Agressive systemic  mastocytosis  M97423: Mast cell leukaemia  M97403: Mast cell sarcoma  NA | Systemic mastocytosis associated with non-mast cell hematopoietic cloning (SM-AHNMD)  Aggressive systemic mastocytosis (ASM)  Mast cell leukemia (MCL)  Mast cell sarcoma (MSC)  Extracutaneous mastocytoma (EM)  NA |

^1^ Patients registered with DD470 and age ≤15 were considered mastocytoma patients, no: 14

^2^ Patients registered with DD470 and age >15 were considered indolent systemic mastocytosis patients, no: 272

^3^ A review of patients registered with M97411 shoved a dominance of appertaining benign ICD10 mastocytosis codes

**Supplementary A:** ICD, SNOMED and corresponding WHO diagnoses, which were used in the identification process of mastocytosis patient in the present study.
