# Supplementary material for: How “benign” is cutaneous mastocytosis? A Danish registry-based matched cohort study
Source: Int J Womens Dermatol. 2020 Jun 1;6(4):294–300. doi: 10.1016/j.ijwd.2020.05.013 (PMC7522902; doi:10.1016/j.ijwd.2020.05.013)
Supplement: Supplementary data 2 [file mmc2.pdf]

Supplementary tables

Table A. Charlson comorbidity index

Weights	Conditions	ICD-8 codes	ICD-10 codes
1	Myocardial infarction	410	I21; I22; I23
	Congestive heart failure	427.09; 427.10; 427.11; 427.19; 428.99; 782.49	I50; I11.0; I13.0; I13.2
	Peripheral vascular disease	440; 441; 442; 443; 444; 445	I70; I71; I72; I73; I74; I77
	Cerebrovascular disease	430-438	I60-I69; G45; G46
	Dementia	290.09-290.19; 293.09	F00-F03; F05.1; G30
	Chronic pulmonary disease	490-493; 515-518	J40-J47; J60-J67; J68.4; J70.1; J70.3; J84.1; J92.0; J96.1; J98.2; J98.3
	Connective tissue disease	712; 716; 734; 446; 135.99	M05; M06; M08; M09; M30; M31; M32; M33; M34; M35; M36; D86
	Ulcer disease	530.91; 530.98; 531-534	K22.1; K25-K28
	Mild liver disease	571; 573.01; 573.04	B18; K70.0-K70.3; K70.9; K71; K73; K74; K76.0
	Diabetes without end organ damage	249.00; 249.06; 249.07; 249.09; 250.00; 250.06; 250.07; 250.09	E10.0, E10.1; E10.9; E11.0; E11.1; E11.9
2	Diabetes with end organ damage	249.01-249.05; 249.08; 250.01-250.05; 250.08	E10.2-E10.8, E11.2-E11.8
	Hemiplegia	344	G81; G82
	Moderate to severe renal disease	403; 404; 580-583; 584; 590.09; 593.19; 753.10-753.19; 792	I12; I13; N00-N05; N07; N11; N14; N17-N19; Q61
	Non-metastatic solid tumour	140-194	C00-C75
	Leukaemia	204-207	C91-C95
	Lymphoma	200-203; 275.59	C81-C85; C88; C90; C96
3	Moderate to severe liver disease	070.00; 070.02; 070.04; 070.06; 070.08; 573.00; 456.00-456.09	B15.0; B16.0; B16.2; B19.0; K70.4; K72; K76.6; I85
6	Metastatic cancer	195-198; 199	C76-C80
	AIDS	079.83	B21-B24
